# From Anxiety to Hopelessness: Examining Influential Psychological Processes Affecting Mental Health Status of Spanish Nurses During the COVID-19 Pandemic

**DOI:** 10.3390/medicina61020236

**Published:** 2025-01-28

**Authors:** Cecilia Peñacoba-Puente, Fernanda Gil-Almagro, Fernando José García-Hedrera, Francisco Javier Carmona-Monge

**Affiliations:** 1 Departamento de Psicología, Facultad de Ciencias de la Salud, Universidad Rey Juan Carlo, 28922 Alcorcón, Madrid, Spain; cecilia.penacoba@urjc.es; 2 Departamento de Enfermería, Universidad Francisco de Vitoria, 28223 Pozuelo de Alarcón, Madrid, Spain; fgilalmagro@gmail.com; 3 Unidad de Cuidados Críticos, Hospital Universitario Fundación Alcorcón, 28922 Alcorcón, Madrid, Spain; fjgarciah@gmail.com; 4 Departamento de Anestesiología, Reanimación y Terapéutica del Dolor, Hospital Universitario Santiago de Compostela, 15706 Santiago de Compostela, A Coruña, Spain

**Keywords:** nursing, hopelessness, cognitive fusion, burnout, professional, anxiety

## Abstract

*Background and Objective:* to test a model aimed at delving into the psychological processes that lead to hopelessness in Spanish nurses two years after a stressful work situation. The model proposed starts with the anxiety experienced at the beginning of the COVID-19 pandemic and includes cognitive fusion and emotional exhaustion, evaluated six months after the end of the confinement, as possible contributing factors to development of hopelessness. *Design:* prospective study with three data collection periods, May to June 2020 (period 1), January to April 2021 (period 2), April to July 2022 (period 3). *Materials and Methods:* The sample (*n* = 131 Spanish nurses) was selected by non-probabilistic convenience sampling. The inclusion criteria were as follows: being female, over 18 years of age, and working as a nurse in direct contact with COVID-19 patients. *Results:* The model proposed was statistically significant (B = 0.34, SE = 0.07, t = 5.15, *p* < 0.001, 95% CI = [0.21, 0.47]), contributing to the explanation of 28% of the variance of hopelessness, finding that the direct anxiety–hopelessness effect was equally significant (B = 0.19, SE = 0.08, t = 2.46, *p* = 0.014, 95% CI = [0.04, 0.34]). An effect of anxiety on cognitive fusion and on emotional exhaustion was observed. In turn, cognitive fusion had a significant effect on emotional exhaustion but not on hopelessness. Finally, emotional exhaustion had a significant effect on hopelessness. In this final model, years of experience had a significant effect (*p* = 0.004) on hopelessness. *Conclusions:* Cognitive fusion and emotional exhaustion are shown to be relevant psychological processes upon which to intervene to contribute to the improvement of the mental health of nurses regarding the COVID-19 pandemic.

## 1. Introduction

More than three years have passed since the beginning of the pandemic caused by COVID-19, declared as the most severe public health crisis the world has faced since the establishment of the World Health Organization (WHO). The consequences on the mental health of healthcare workers (HCWs) who were on the front line during the so-called first waves have been extensively reported in the literature [1,2]. Based on the knowledge of these negative effects, current prospective studies raise two questions of particular interest in this context: on the one hand, whether these effects observed in the first waves of the pandemic are still maintained years later and, on the other hand, to what extent certain psychosocial variables may constitute risk or protective variables in relation to the impact on mental health of highly stressful stimuli, such as in the case of the COVID-19 pandemic. The present study aims to shed some light on the latter issue.

Hopelessness is a central variable linked to mental processes, particularly depression [3]. Hopelessness, along with helplessness, has been extensively studied in the explanation of depressive disorders, within the so-called helplessness–hopelessness perspective [4]. Beck defined hopelessness as a negative outlook toward the future, with depressive affect stemming from negative cognitions. This concept relates closely to helplessness, which describes a perceived lack of control over circumstances [5]. In highly stressful contexts, such as the COVID-19 pandemic [1,2], these processes are expected to appear in HCWs. Previous studies have demonstrated a significant link between anxiety at the onset of the pandemic and hopelessness, especially among nurses, who reported higher levels of stress compared to other HCWs [6]. However, there is limited evidence on the psychological mechanisms that influence this relationship. Previous studies point to a relationship between hopelessness and burnout in healthcare workers during the pandemic, showing that training in emotional intelligence contributes to a reduction in both variables [7].

Although the importance of cognitive aspects has been emphasized [5], they have hardly been analyzed in prospective models, and in HCWs in particular [8]. This is why this research focuses on hopelessness, two years after the onset of the COVID-19 pandemic, starting with the anxiety generated in the early stages and proposing cognitive and emotional variables as possible mediators.

A review of the literature on hopelessness in HCWs during the COVID-19 pandemic highlights the relevance of this variable. As has been pointed out, hopelessness in healthcare workers throughout the COVID-19 pandemic has been related to depression [9] and to altered sleeping and eating patterns [9]. A close relationship has also been found between hopelessness and burnout in nursing [7,10].

Underpinning hopelessness in the COVID-19 pandemic, studies in HCWs emphasize the role of anxiety [11,12]. Furthermore, along with anxiety, other studies have suggested the possible influence of social isolation, aggravated during the pandemic, as a factor that can increase hopelessness [13,14]. Similarly, previous studies have identified helplessness as a possible precursor of hopelessness, establishing both the relationship and the differentiation between the two variables [13]. In fact, helplessness has been a highly studied concept among healthcare workers [15,16], particularly in nurses during the COVID-19 pandemic [13]. Helplessness has been linked to anxiety, shiftwork and depression.

However, while previous literature has provided evidence for the role of anxiety associated with the onset of the COVID-19 pandemic [17] as a possible antecedent of hopelessness, little is known about the psychological processes influencing this relationship. In other contexts, how coping with stressful situations [18] and personality play a relevant role in the prediction of hopelessness has been analyzed. In particular, cognitive flexibility, intolerance of uncertainty [19] and sense of coherence [20] have been found to act as protective traits, whereas neuroticism can act as a risk factor [21]. However, as noted, to the best of our knowledge, studies on the psychological variables associated with hopelessness have not been carried out among HCWs during the COVID-19 pandemic.

Two processes have garnered attention in their relationship with hopelessness: emotional exhaustion and cognitive fusion. Inadequate management of anxiety can lead to burnout, especially among healthcare personnel and nurses [22,23]. Research during COVID-19 and previous pandemics, such as SARS-CoV-2 (2016–2021), has highlighted emotional exhaustion as a key component of burnout syndrome [24,25,26,27]. Personality variables, such as cognitive fusion, also play a critical role in the stress–burnout relationship. According to Lazarus’ transactional model of stress [28], burnout arises from the interaction between situational demands and individual characteristics [29]. Among psychosocial variables, like self-efficacy, optimism, and affectivity [30], cognitive fusion stands out for its negative impact on mental health [31,32]. Defined as perceiving thoughts as identical to events [33], cognitive fusion contributes to ineffective stress management, exacerbating anxiety, which can escalate into chronic emotional exhaustion [34,35].

In healthcare personnel, cognitive fusion has been shown to be a clear negative factor associated with increased mental fatigue [36]. However, despite its interest, this variable has hardly been studied in the context of the COVID-19 pandemic, although the few existing studies in the healthcare community show that high levels of cognitive fusion are associated with negative socioemotional consequences in the medium to long term [37]. In particular, studies carried out in nurses indicate that high values of cognitive fusion are associated with mental health problems; it seems as though the thoughts themselves become stressors because of the tendency to become entangled in them and the inability to interpret them for what they are, which is simply thoughts [37].

Although previous literature points to the relevance of studying hopelessness as a key mood state associated with quality of life [12] or suicidal ideation [10], few studies have assessed this variable in healthcare personnel during the COVID-19 pandemic. In this context, to contribute to the explanation of hopelessness (11 April to 15 July 2022), the model proposed in the present study in Spanish nurses starts from the anxiety experienced at the beginning of the pandemic (5 May to 21 June 2020), including cognitive fusion and emotional exhaustion evaluated six months later (9 January to 9 April 2021) as possible contributing factors to the development or chronification of hopelessness.

In particular, cognitive fusion is proposed as a non-adaptive mechanism to relate especially to the cognitive components (i.e., rumination) of the anxiety generated at the beginning of the pandemic [34], which in turn can generate greater emotional exhaustion [38], eventually triggering, in the medium to long term, a feeling of hopelessness.

To explore this issue further, the present study delves into the relationship between anxiety and hopelessness, considering emotional exhaustion (as an essential component of the burnout syndrome) and cognitive fusion as mediating variables. The first of these variables has received considerable attention in research on mental health in the healthcare community during the pandemic [39,40], while there are few published studies on the latter.

## 2. Materials and Methods

Design: This is a prospective study with three data collection periods: 5 May to 21 June 2020 (period 1), 9 January to 9 April 2021 (period 2) and 11 April to 15 July 2022 (period 3). During the first data collection period, Spain was in COVID-19 confinement, the number of positive cases amounting to 213,435 with 24,543 deaths recorded. During the second data collection period, the health situation continued to be very complicated, with 3,275,819 positive COVID-19 cases recorded during the month of March. In the third period, Spain was amid a vaccination campaign and was currently administering the second dose of vaccine against COVID-19. The number of infected persons as of 17 June 2022 was 12,563,399, with 107,482 confirmed deaths due to COVID-19.

### 2.1. Procedure and Participants

Data collection was carried out by means of an online electronic questionnaire designed for this purpose by the research team. The objective of the study was presented at the beginning of the questionnaire, and informed consent was requested to use the data of the participants in the study. The sample was selected using non-probabilistic convenience sampling, sending the link to nurses in the Spanish healthcare system who had been in contact with patients affected by COVID-19 and disseminating it through social networks (Facebook, Twitter, LinkedIn and WhatsApp), as well as through corporate e-mails from public and private healthcare services in the Spanish system. For the dissemination of the evaluation protocol at the second and third time points, the e-mails of the nurses who had participated in the first time point were used, requesting, once again, their participation in the following phases of the study.

The inclusion criteria were as follows: female, over 18 years of age, and working as a nurse in direct contact with COVID-19 patients. Exclusion criteria were change of service during the data collection periods and working as a nurse manager.

A sample size of *n* = 120 is considered for prospective mediation studies [41]. The final sample of the study was 131 nurses. In the first time period, 657 nurses participated, reducing to 490 nurses in the second time period. This sample loss is common in prospective studies in HCWs, especially in research conducted during the COVID-19 pandemic [42,43].

### 2.2. Variables and Instruments

#### 2.2.1. Sociodemographic and Occupational Variables

Sociodemographic data (age, cohabitation situation) and occupational data (employment status, degree, work shift and years of experience as nurse) were collected. An ad hoc questionnaire prepared by the research team was used to collect these data.

#### 2.2.2. Outcome Variables

Generalized anxiety disorder [assessed at period 1]: The presence of symptoms of generalized anxiety disorder was assessed using the 7-item self-rated Generalized Anxiety Disorder Scale (GAD-7) [44], Spanish version [45]. The scale is composed of 7 items with a 4-point Likert response format ranging from 0 (not at all) to 3 (nearly every day). Higher scores on the scale are indicative of greater severity of symptoms. In the present study, an excellent internal consistency was obtained (α = 0.93).

Cognitive fusion [assessed at period 2]: The Spanish version [46] of the Cognitive Fusion Questionnaire (CFQ) [47] was used. Consisting of 7 items that assess cognitive fusion (the extent to which we are psychologically entangled with or dominated by the form and content of our own thoughts), it consists of a Likert-type response scale with 7 response alternatives, ranging from 1 (never) to 7 (always). Cronbach’s alpha was 0.97 in our study.

Emotional Exhaustion [assessed at period 2]: The Maslach Burnout Inventory–Human Services Survey (MBI–HSS; [48] Spanish version [49]) was used. This 22-item scale adopts a 7-point Likert response format ranging from 0 (never) to 6 (everyday). The instrument assess three burnout dimensions or subscales (i.e., depersonalization, emotional exhaustion, diminished self-fulfillment). The emotional exhaustion dimension was used for the present study (i.e., feelings of depletion and overloading of one’s emotional and physical resources; 9 items). Adequate internal consistency was found (α = 0.91) in our data. 

Hopelessness [assessed at period 3]: The Beck Hopelessness Scale [50] was used in its Spanish version [51]. The scale is designed to measure the cognitive, affective, and motivational dimensions of hopelessness using a 20-item format scored on a true–false rating scale; the higher the scores, the greater the hopelessness levels. Cronbach’s α in our sample was 0.87.

### 2.3. Data Analysis

**Figure 1:**
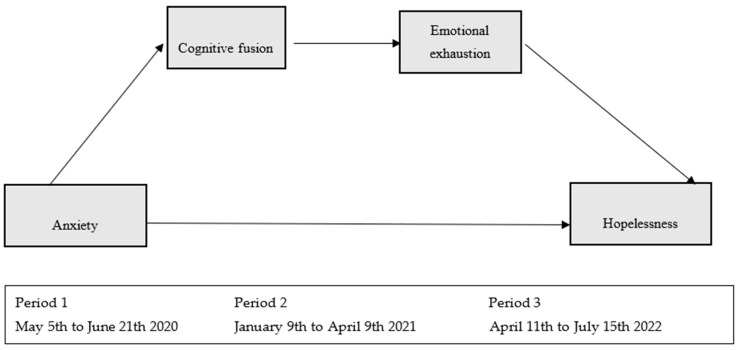
Proposed theoretical model.

Descriptive and Cronbach’s alpha analysis were performed. Qualitative variables were described with frequencies (*n*) and percentages (%) and quantitative variables with mean (*M*) and standard deviation (*SD*). To analyze the bivariate association between variables (analysis of possible covariates), Student’s *t* test, one-factor analysis of variance (ANOVA) and Pearson’s correlation were used, depending on the nature of the variables analyzed. Different multivariate regressions were carried out using the PROCESS macro, specifically through a multiple mediation analysis with two mediating variables forming a causal chain (model 6). In this model, anxiety assessed at the first time period was the predictor variable (X), cognitive fusion (M1) and emotional exhaustion (M2) assessed six months later as mediating variables (second time period) and hopelessness two years after the onset of the pandemic (third time period) as the outcome variable (Y) (see Figure 1). Statistical analysis was carried out with Statistical Package for the Social Sciences (SPSS) version 21 for Windows. The results were considered statistically significant for values of *p* < 0.05.

Figure 1 presents the theoretical model proposed in the study, which explains the relationship between anxiety, experienced at the beginning of the COVID-19 pandemic, and hopelessness assessed two years later. This model introduces cognitive fusion and emotional exhaustion, measured six months later, as mediating variables in a causal chain. Anxiety directly influences cognitive fusion, a non-adaptive process in which thoughts are perceived as unchanging realities, increasing rumination and the inability to handle stress. In turn, cognitive fusion contributes to emotional exhaustion, a key component of burnout characterized by physical and emotional exhaustion in the face of the workload. Finally, emotional exhaustion emerges as a crucial factor predicting high levels of hopelessness, understood as a negative perception of the future and a loss of control over circumstances. The model explains 28% of the variance of hopelessness, highlighting the mediating role of emotional exhaustion, while cognitive fusion acts indirectly through this process.

## 3. Results

### 3.1. Characteristics of the Sample

The average age of the participants (*n* = 131) was 40.98 (SD = 9.43) with a minimum age of 21 and a maximum age of 62. The average years of experience as a nurse was 11, with a minimum of 0 and a maximum of 35. Most of the sample lived with a partner (67.2%, *n* = 88).

Regarding distribution by services, the majority of the sample was either in the ICU (41.2%, n = 54) or in hospitalization (33.6%, *n* = 44); nurses belonging to the emergency department accounted for 15.3% (n = 20) and 9.9% (*n* = 13) were in primary care (PC) or consultation rooms. Regarding employment status, the highest percentage of the sample was permanently employed (51.1%, *n* = 67), the rest of the sample being either interim (29.8%, *n* = 39) or temporary (19.1%, *n* = 25). Almost half of the sample had additional higher education (Master, PhD; 41.2%, *n* = 54) to what is necessary for the performance of their profession. Regarding work shifts, most of the sample had rotating shifts (53.4%, *n* = 70) and shifts longer than 10 h (28.3%, *n* = 37). The rest of the nurses had fixed morning, afternoon or night shifts (16.8%, *n* = 22), or worked fixed shifts plus on-call (1.5%, *n* = 2).

### 3.2. Descriptive Statistics and Correlations Between the Variables of Interest

Table 1 shows the descriptive statistics and correlations between the variables included in the proposed model. Taking into account the classification of anxiety levels [45] (0–5: minimal/no anxiety, 6–10: mild anxiety, 11–15: moderate anxiety, 16–21: severe anxiety), it was found that 61.1% of the sample had moderate and severe levels. The mean values for cognitive fusion were considered moderate. For emotional exhaustion, the scores can be considered moderately high. Finally, the mean for hopelessness was below the theoretical range of the scale. It is interesting to note that, for all variables, the theoretical range coincided with the sample range, which implies, in relation to the risk profiles, that some participants had reached the maximum scores on the scales. The correlation analysis showed significant positive relationships between all variables (*p* < 0.01), with a particularly high effect size for the correlation between cognitive fusion and emotional exhaustion (*r^2^* = 0.70), followed by the correlations between anxiety and emotional exhaustion (*r^2^* = 0.57) and between anxiety and cognitive fusion (*r^2^* = 0.50).

### 3.3. Relationship Between Outcome Variables and Sociodemographic and Occupational Variables—Covariate Analysis

Regarding the analyses of the relationships between the variables proposed in the model (anxiety, cognitive fusion, emotional exhaustion, hopelessness) and age, service, cohabitation situation, employment status, educational level, work shift and years of experience as a nurse, we found statistically significant (negative) relationships between years of experience as a nurse and anxiety (*r^2^* = −0.264, *p* = 0.003), cognitive fusion (*r^2^* = −0.225, *p* = 0.10) and emotional exhaustion (*r^2^* = −0.242, *p* = 0.006). Regarding the rest of the variables analyzed, no statistically significant differences were observed. Based on these results, the multiple mediation model proposed includes years of experience as a nurse as a covariate.

### 3.4. Multiple Mediation Analysis—Prediction of Hopelessness (Period 3) Starting with Anxiety as Antecedent (Period 1) and Proposing Cognitive Fusion and Emotional Exhaustion (Period 2) as Mediating Factors

Table 2 shows the regression analyses performed to predict hopelessness through anxiety experienced at the onset of the pandemic, using cognitive fusion (M1) and emotional exhaustion (M2) as mediating variables assessed six months after the onset of the pandemic.

As can be seen in Table 2, after controlling for the possible effect of the covariate (years of experience as a nurse), the mediation results showed that the model explained 28% of the variance of hopelessness two years after the anxiety suffered at the beginning of the pandemic (F = 9.71, *p* < 0.001). The total model effect was significant (B = 0.34, SE = 0.07, t = 5.15, *p* < 0.001, 95% CI = [0.21, 0.47]). The effect of anxiety on hopelessness was mediated by cognitive fusion and emotional exhaustion, with the direct anxiety–hopelessness effect being equally significant (B = 0.19, SE = 0.08, t = 2.46, *p* = 0.014, 95% CI = [0.04, 0.34]).

Analyzing the effects in detail, an effect of anxiety on cognitive fusion (B = 0.92; SE = 0.15; 95% CI = [0.62, 1.23]) and on emotional exhaustion (B = 0.67; SE = 0.16; 95% CI = [0.34, 0.99]) was observed. In turn, cognitive fusion had a significant effect on emotional exhaustion (B = 0.67; SE = 0.08; 95% CI = [0.51, 0.84]) but not on hopelessness (B = 0.02; SE = 0.05; 95% CI = [−0.07, 0.11]). Finally, emotional exhaustion had a significant effect on hopelessness (B = 0.10; SE = 0.04; 95% CI = [0.02, 0.18]). Anxiety contributed to the explanation of 26% of cognitive fusion, and anxiety and cognitive fusion contributed to 57% of the variance of emotional exhaustion.

**Figure 2:**
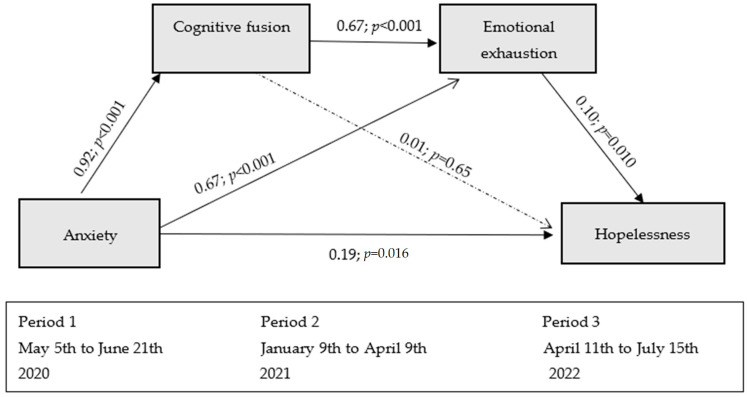
Serial mediation model of the explanation of hopelessness based on anxiety (cognitive fusion and emotional exhaustion as mediators).

Taken together, the moderated mediation model with two mediators (cognitive fusion and emotional exhaustion) contributed to the explanation of 28% of the variance of hopelessness from anxiety assessed two years earlier. In this final model, years of experience had a significant effect (*p* = 0.004) on hopelessness, contributing positively to increased hopelessness across the variables in the model (B = 0.12; SE = 0.04; 95% CI = [0.04, 0.21]) (see Figure 2).

## 4. Discussion

The present study aimed to delve into the psychological processes that may have contributed to hopelessness (two years later) because of the anxiety experienced by nurses at the onset of the COVID-19 pandemic. Within the hypothesized model, cognitive fusion and emotional exhaustion experienced six months after the beginning of the pandemic are proposed as possible contributing factors to its development.

The bivariate analyses (correlations) performed initially support the significant and positive relationships established between the variables proposed in the model. The correlation observed between cognitive fusion and emotional exhaustion is particularly relevant due to its magnitude. Although studies are very scarce, previous research has highlighted the relationship between cognitive fusion and burnout, and specifically emotional exhaustion [52,53]. Our results support the findings from previous literature and provide new data, due to the design of the study and the sample used. Specifically, our results highlight the close relationship between cognitive fusion and emotional exhaustion assessed in nurses six months after the onset of the pandemic. The results also show significant and positive correlations between anxiety and cognitive fusion. As noted, previous literature has highlighted the close relationship between these variables [35,54]. What is particularly novel in the present study is that the relationship between anxiety and cognitive fusion is maintained, in this case, in nurses in contact with COVID-19 patients, with six months between the assessment of these variables. Finally, it is worth noting the significant and positive relationships that hopelessness maintains with anxiety, cognitive fusion and emotional exhaustion, with time periods between six months and two years of difference between the measures. The relationship between anxiety and hopelessness has been justified throughout the literature [55,56], with research in nurses in particular showing that elevated levels of anxiety lead to hopelessness [11]. Similarly, although to a lesser extent, previous literature has shown relationships between emotional exhaustion and hopelessness [10,14], and this relationship has also been analyzed in nurses [57]. In the context at hand, it should be noted that pandemic-related emotional exhaustion in health professionals has been found to correlate with hopelessness [14]. Finally, the relationship between hopelessness and cognitive fusion has hardly been explored [58]; specifically, in the aforementioned research [58], hopelessness is controlled for, in order to analyze the interaction between cognitive fusion and the perception of loneliness and burden, within the interpersonal theory of suicide. In the absence of studies in this area, the inclusion of this variable in the present study can be considered a particularly novel aspect.

In this context, as noted above, the main contribution of the present study lies, in our opinion, in the development of a tested model that includes cognitive fusion and emotional exhaustion as psychological processes involved in the relationship between anxiety experienced at the beginning of the pandemic and hopelessness two years later, establishing an intermediate measurement period. The results show that the proposed model is significant and that, as a whole, it explains 28% of the variability of hopelessness. Significant direct effects of anxiety on hopelessness are observed, in line with previous literature [11,12], but providing the advantages of a prospective study. However, it is necessary to highlight that the explanatory power of anxiety over hopelessness increases significantly when the two mediators (cognitive fusion and emotional exhaustion) are added.

Despite the special interest in recent years in the negative health effects of cognitive fusion within cognitive flexibility models [31,32], surprisingly little research has been carried out on this in the context of the COVID-19 pandemic, and in particular in HCWs. The few studies carried out on nurses during the COVID-19 pandemic point out cognitive fusion as a risk factor because of its associations with mental fatigue or emotional symptoms [36]. It has been found that certain intervention strategies aimed at reducing cognitive fusion can provide nurses with effective coping strategies that contribute to the reduction of mental health problems and decrease sleep problems that appear in the face of work stressors [37]. The model proposed in this study, starting with the anxiety observed at the onset of the COVID-19 pandemic, and its non-adaptive coping through cognitive fusion processes, shows, in turn, how cognitive fusion can trigger emotional exhaustion six months after the onset of the pandemic, ultimately contributing to higher levels of hopelessness two years later. The importance of emotional exhaustion as a particularly distressing sign of burnout has already been highlighted in previous literature [59], especially in nurses [60] and in particular during the COVID-19 pandemic [61]. Likewise, previous research has pointed out the relationship between emotional exhaustion and hopelessness [14]. The proposed model, therefore, allows the integration of processes studied to a greater (i.e., emotional exhaustion) or lesser extent (i.e., cognitive fusion) in HCWs during the pandemic, to contribute to an explanation of hopelessness through a prospective design, which allows us to delve into the long-term consequences of a particularly adverse situation experienced by nurses.

Although years of professional experience and their relationship with hopelessness has not been previously studied, their relationship with other psycho-emotional consequences suffered after a stressful work event has been described in the existing literature. Professional nursing experience not only provides technical and coping skills, but also emotional strategies that mitigate the impact of stressful work events, such as those experienced during the pandemic [62]. Several studies have pointed out that lack of experience is associated with higher levels of burnout and hopelessness, especially in situations of high workload and chronic stress [63]. In the particular case of Spain, the shortage of nurses and the aging of the professional workforce have generated a more demanding work environment, with a significant increase in the work overload of young and less experienced nurses [64]. This could explain, in part, the higher levels of hopelessness observed in this group, as the lack of adequate resources and support amplifies the negative impact of work stress. In this sense, the importance of implementing retention and training policies that allow nurses to gain experience and have adequate organizational support, thus mitigating the adverse effects of the work context on mental health, is emphasized [65,66].

Within the proposed model, it is interesting to note that cognitive fusion by itself has no direct effect on hopelessness. That is, in the proposed serial mediator model (cognitive fusion-emotional exhaustion), the role of emotional exhaustion is necessary to produce hopelessness. Therefore, cognitive fusion plays an important role between anxiety and emotional exhaustion, but not between anxiety and hopelessness. Although, to our knowledge, there are no previous data to explain these results, it could be suggested that cognitive fusion acts as a non-adaptive (in this case cognitive) coping strategy in the face of anxiety processes [35,54], contributing to their chronification (becoming entangled in one’s own thoughts) [33] and to an increase in burnout symptoms (in this case emotional exhaustion [52,53]). In other words, cognitive fusion could be understood as a maladaptive coping strategy in stress processes [36]. However, cognitive fusion does not have a direct effect on hopelessness. Hopelessness, linked to this perception of uncontrollability, of helplessness, in situations of anxiety, seems to be associated more with highly stressful emotional processes, with the impossibility of regulating emotions, such as emotional exhaustion [67].

This study has several practical implications that should be considered. Given the relevance of reducing emotional exhaustion derived from anxiety produced by a work stressor, it seems necessary to establish preventive strategies. The results of the covariate analysis show that nursing experience has a significant negative relationship with emotional exhaustion and anxiety. Therefore, after reviewing the previous literature, and taking into account the skills that experience provides, it is suggested that a key measure to prevent anxiety derived from a traumatic work event from generating emotional exhaustion would be to keep nursing professionals trained and updated, thus generating the same competencies that an experienced nurse may have and contributing to the prevention of emotional exhaustion due to anxiety at work [68]. Likewise, as a preventive strategy, the establishment of programs could be proposed aimed at reducing cognitive fusion, given that our findings show that anxiety that leads to cognitive fusion causes an increase in emotional exhaustion and with it an increase in hopelessness. To our knowledge, no interventions in HCWs aimed at decreasing cognitive fusion have been found. However, the literature regarding its approach in other settings [69,70] suggests interventions from Acceptance and Commitment therapy, applying mindfulness techniques in particular. However, further research is needed in relation to these techniques, and in particular to the group we are dealing with [71].

In short, at an applied level, future lines of research could be directed to the development of interventions that allow an adaptive management of anxiety by HCWs and nurses, including the management of cognitive fusion as a risk factor, and consequently reducing emotional exhaustion and hopelessness derived from work-related traumatic events.

### Limitations

The present study has several limitations to be considered. First is the convenience sample, which implies caution in generalizing the results. It can also be considered a bias not to have considered anxiety symptoms prior to the study (before the pandemic began). Finally, although one of the strengths of the present study is its prospective nature, additional temporal evaluation measures could have been incorporated between the three established time periods.

## 5. Conclusions

Despite the above limitations, the results of our study allow us to reflect on the need to establish measures aimed at protecting the psycho-emotional health of nurses. Relevant results regarding the appearance of hopelessness two years after the onset of the pandemic were also found. It should be taken into account that hopelessness is related to the risk of suicide [10], so it is particularly relevant to delve into the factors that may influence hopelessness to increase or become chronic after a traumatic event. The present study reveals the role of cognitive fusion and emotional exhaustion as key psychological processes upon which to intervene in order to contribute to the improvement of the mental health of our healthcare professionals, specifically of nurses.

## Figures and Tables

**Table 1:** Descriptive statistics and correlations between the variables of interest.

	Level (n, %) ^a^	Mean (SD)	Sample Range	Theoretical Range	2	3	4
1. Anxiety	minimal (15, 11.5%) mild (36, 27.5%) moderate (41, 31.3%) severe (39, 29.8%)	11.42 (5.69)	0–21	0–21	0.50 **	0.57 **	0.41 **
2. Cognitive fusion		22.94 (11.07)	7–49	7–49		0.70 **	0.35 **
3. Emotional Exhaustion		28.44 (13.40)	0–54	0–54			0.44 **
4. Hopelessness		5.49 (4.62)	0–20	0–20			

** *p* < 0.01; ^a^ Classification of anxiety levels: minimal/no anxiety (0–5), mild anxiety (6–10), moderate anxiety (11–15), or severe anxiety (16–21).

**Table 2:** Multiple mediation model. Effects of anxiety (antecedent) on hopelessness (consequence) through cognitive fusion and emotional exhaustion (mediators).

Effects of anxiety on cognitive fusion						
VD: Cognitive fusion	B	SE	t	*p*	LLCI	ULCI
VI: Anxiety	0.92	0.15	5.95	<0.001	0.62	1.23
* Experience (years)	−0.11	0.10	−1.14	0.26	−0.31	0.08
	R = 0.51	R^2^ = 0.26	F = 14.77	*p* < 0.001		
Effects of anxiety on emotional exhaustion mediated by cognitive fusion						
VD: Emotional exhaustion	B	SE	t	*p*	LLCI	ULCI
VI: Anxiety	0.67	0.16	4.07	<0.001	0.34	0.99
M1:Cognitive fusion	0.67	0.08	8.06	<0.001	0.51	0.84
* Experience (years)	−0.09	0.09	−0.92	0.36	−0.27	0.10
	R = 0.75	R^2^ = 0.57	F = 40.31	*p* < 0.001		
Effects of anxiety on hopelessness mediated by cognitive fusion and emotional exhaustion						
VD: Hopelessness	B	SE	t	*p*	LLCI	ULCI
VI: Anxiety	0.22	0.08	2.90	0.005	0.071	0.378
M1: Cognitive fusion	0.02	0.05	0.450	0.653	−0.070	0.111
M2: Emotional exhaustion	0.105	0.040	2.62	0.010	0.026	0.184
* Experience (years)	0.123	0.042	2.95	0.004	0.040	0.206
	R = 0.53	R^2^ = 0.28	F = 9.71	*p* < 0.001		

Time evaluation period (in parentheses) of psychological variables: Anxiety (Period 1: 5 May to 21 June 2020), Cognitive fusion (Period 2: 9 January to 9 April 2021), Emotional exhaustion (Period 2: 9 January to 9 April 2021), Hopelessness (Period 3: and 11 April to 15 July 2022). * indicates covariates included in the model.

## Data Availability

Research data will be made available upon request to the corresponding author.
